# Supplementary material for: Corticonigral projections recruit substantia nigra pars lateralis dopaminergic neurons for auditory threat memories
Source: Nat Commun. 2025 Sep 25;16:8384. doi: 10.1038/s41467-025-63132-8 (PMC12462460; doi:10.1038/s41467-025-63132-8)
Supplement: Supplementary file 1 — Supplementary Information [file 41467_2025_63132_MOESM1_ESM.docx]

**
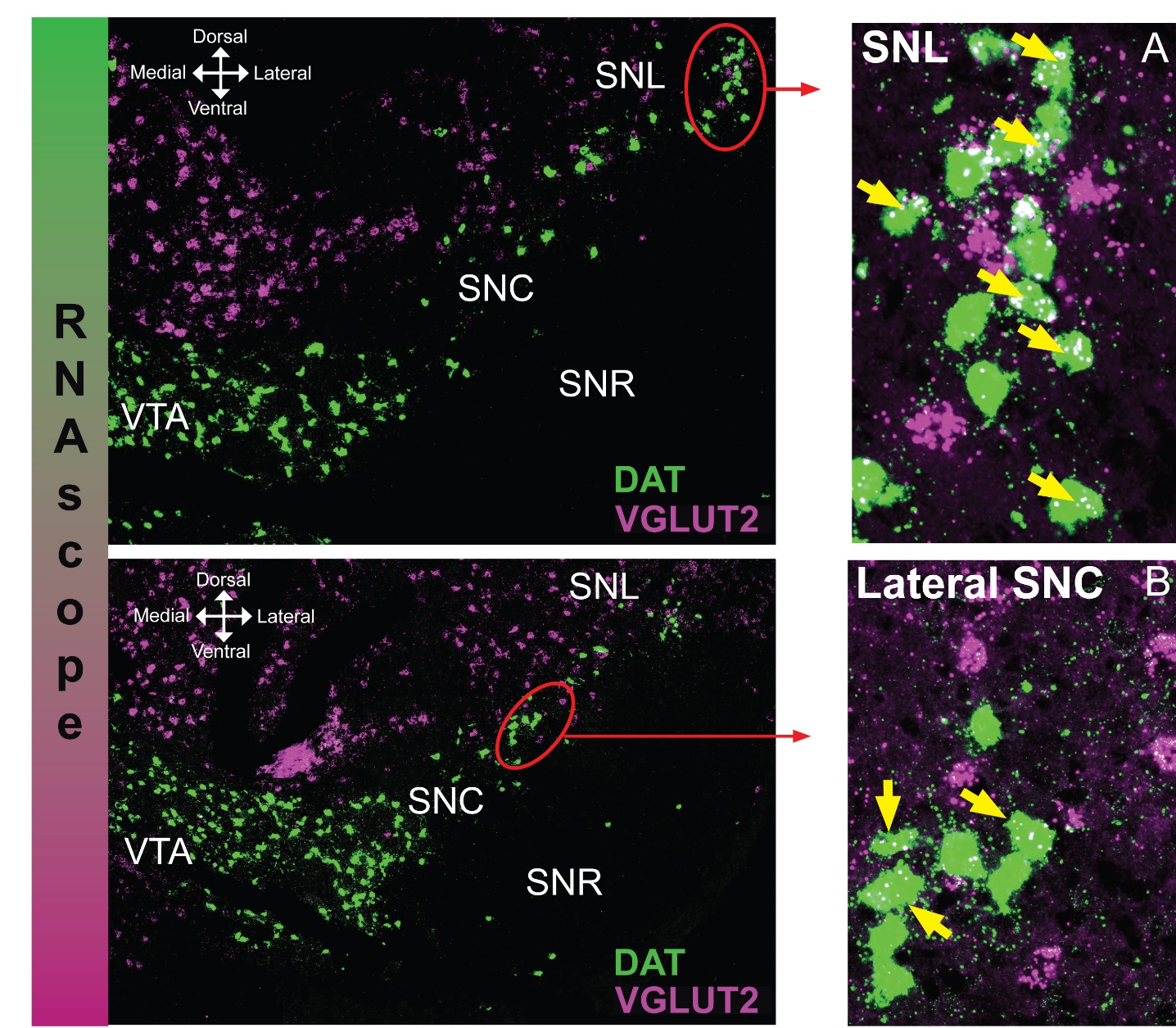
Supplementary Figures**

**Supplementary Figure 1 I VGluT2-Positive Dopaminergic Neurons are located in Substantia Nigra Pars Compacta and Substantia Nigra Pars Lateralis.** *Left*, representative image from in situ hybridization experiment showing a coronal section of substantia nigra from a C57WT mouse with DAT+ (green) and VGluT2+ (purple) neurons. *Right,* Yellow arrows indicate doubly positive neurons for DAT and VGluT2 in SNL (A, top) and lateral SNC (B, bottom).


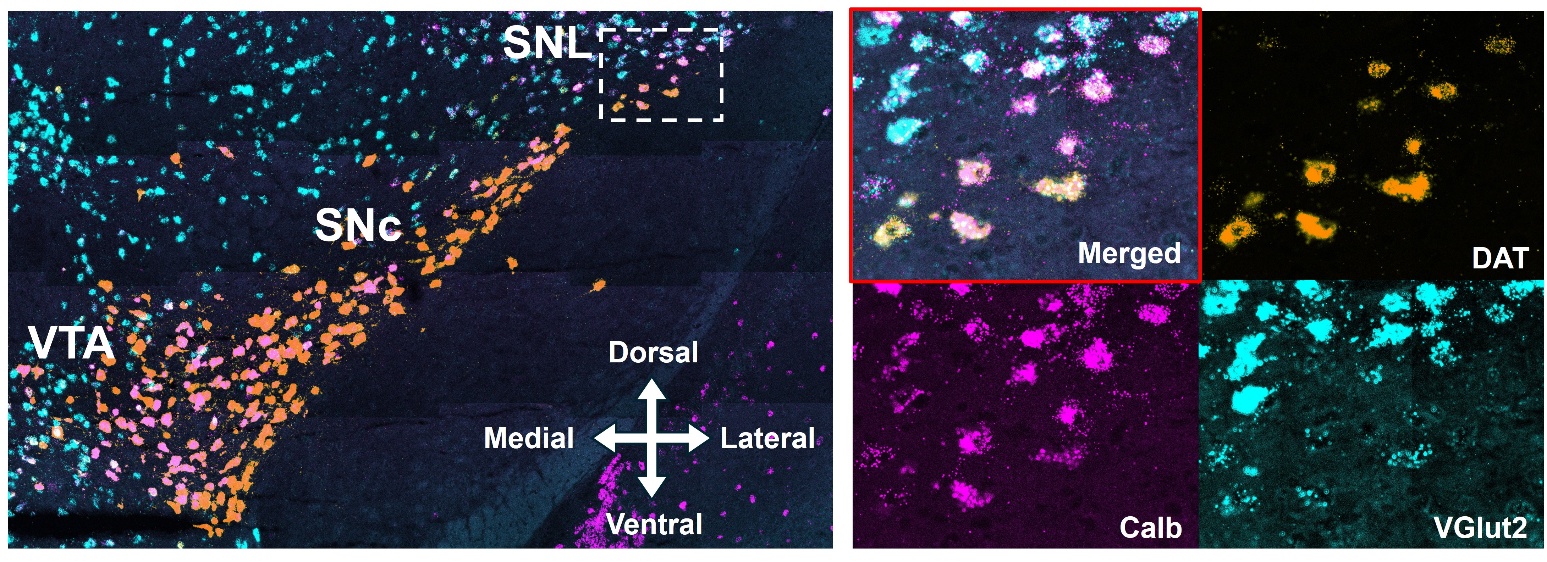


**Supplementary Figure 2 I Dopaminergic Neurons in the Substantia Nigra Pars Lateralis are positive for Calbindin and VGluT2.** Representative image from in situ hybridization experiment showing a substantia nigra coronal section from a C57WT mouse with DAT+ (orange), Calbindin+ (magenta) and VGluT2+ (blue) neurons.


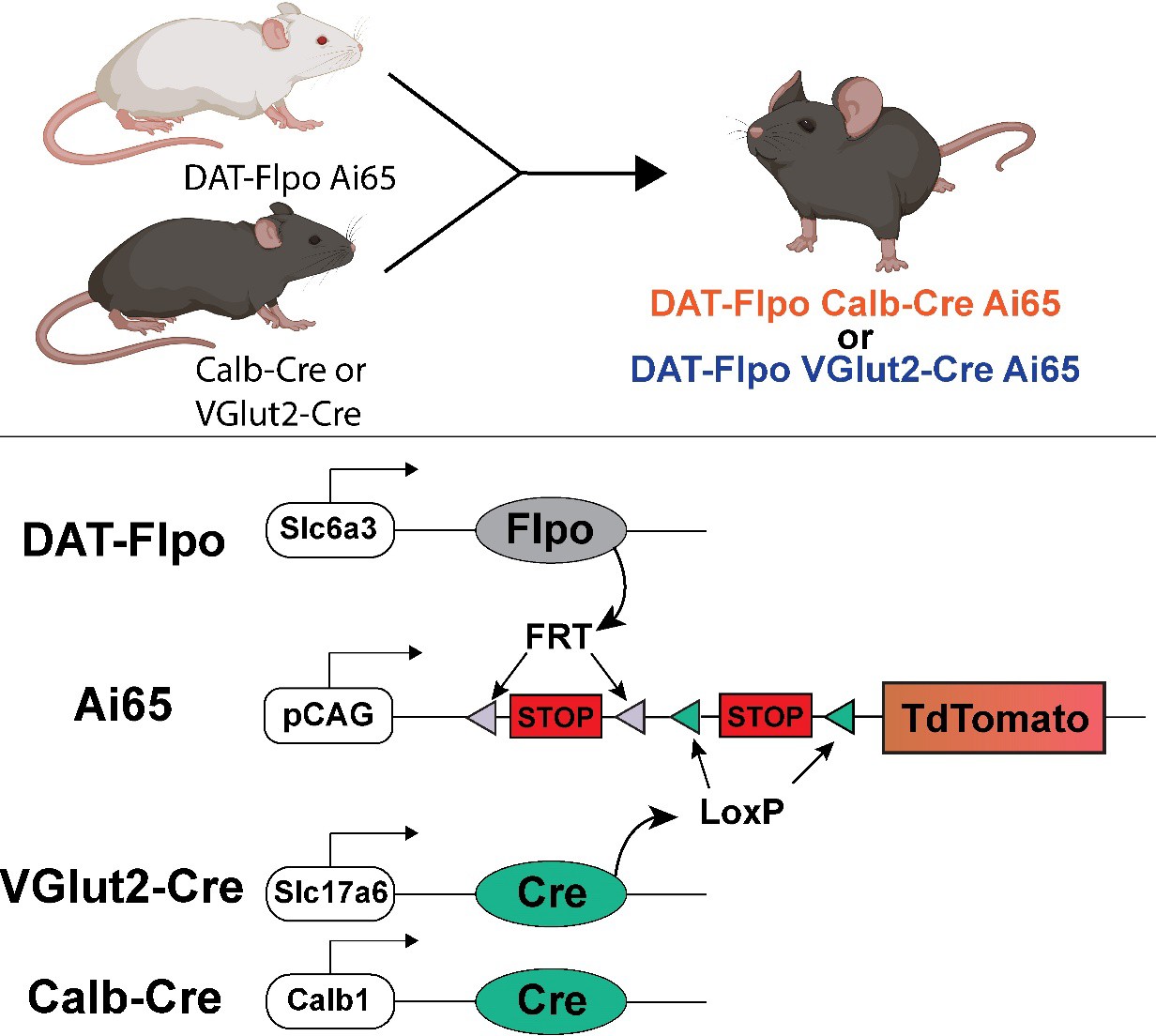


**Supplementary Figure 3 I Intersectional Genetic Strategy to generate DAT-Flp Calb-Cre or DAT-Flp VGluT2-Cre mice. a,b** Intersectional genetic strategy used to generate DAT-Flp Calb-Cre or DAT-Flp VGluT2-Cre mice. Created in BioRender. Sansalone, L. (2025) <https://BioRender.com/lnw5xiq>.


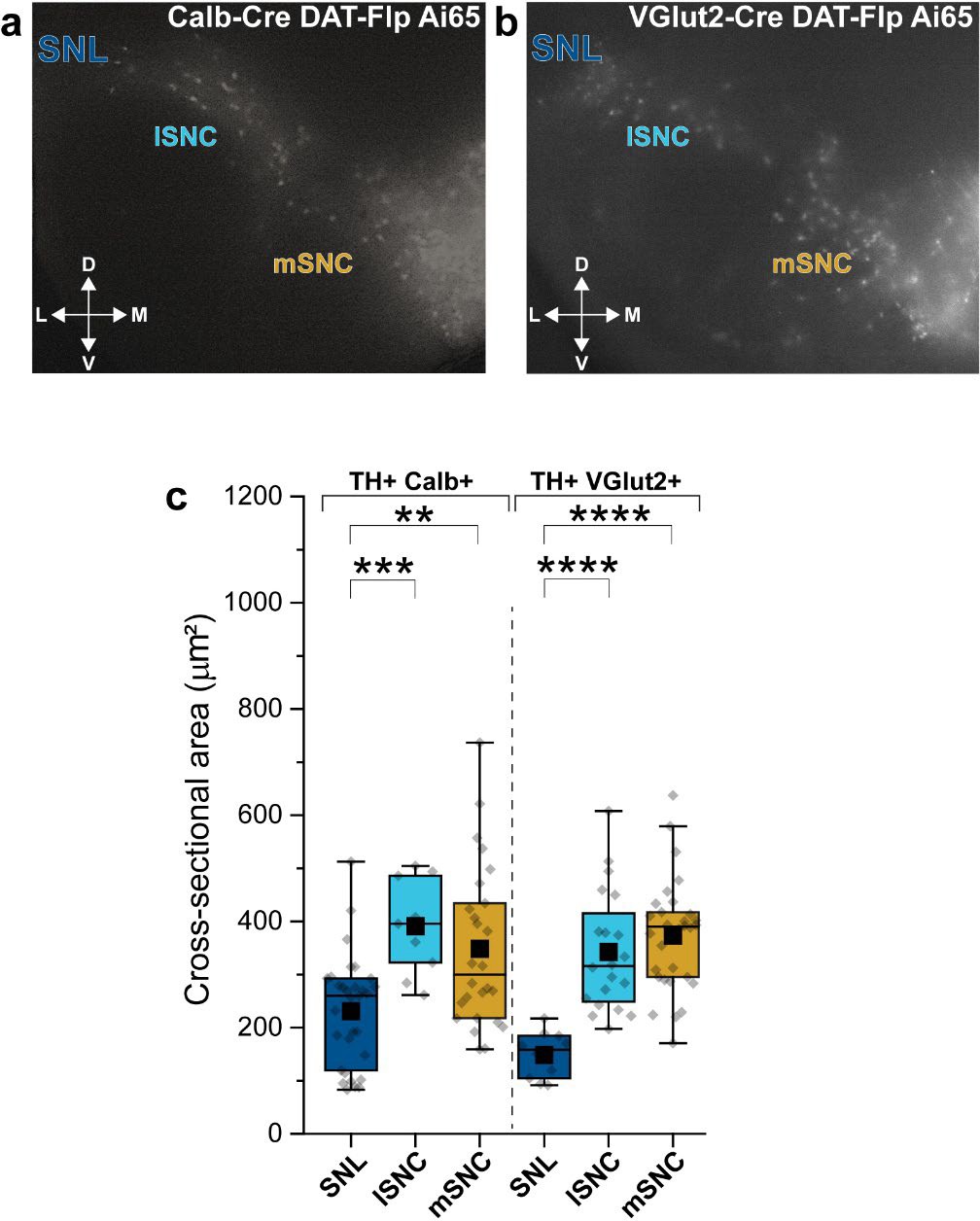


**Supplementary Figure 4 I Dopaminergic Neurons in Substantia Nigra Pars Lateralis are smaller than the ones in Substantia Nigra Pars Compacta. a,b** Representative coronal sections from Calb-Cre DAT-Flp Ai65 or VGluT2-Cre DAT-Flp Ai65 mice showing Calb+ (**a**) or VGluT2+ (**b**) DANs (white) along the mediolateral axis of the substantia nigra. **c.** Bar plots showing cross-sectional somatic areas from TH+/Calb+ neurons (SNL (n=31) vs lSNC (n=9), p = 3.55 x 10^-4^; SNL (n=31) vs mSNC (n=26), p = 0.0056; two-sided unpaired Mann-Whitney) or TH+/VGluT2+ neurons (SNL (n=10) vs lSNC (n=20), p = 31.33 x 10^-7^; SNL (n=10) vs mSNC (n=30), p = 1.65 x 10^-8^; two-sided unpaired Mann-Whitney). Box whiskers represent 25-75% percentiles, solid squares are mean value, horizontal box lines represent medians. ******p <* 0.05, *******p <* 0.01, ********p <* 0.001, *********p <* 0.0001.


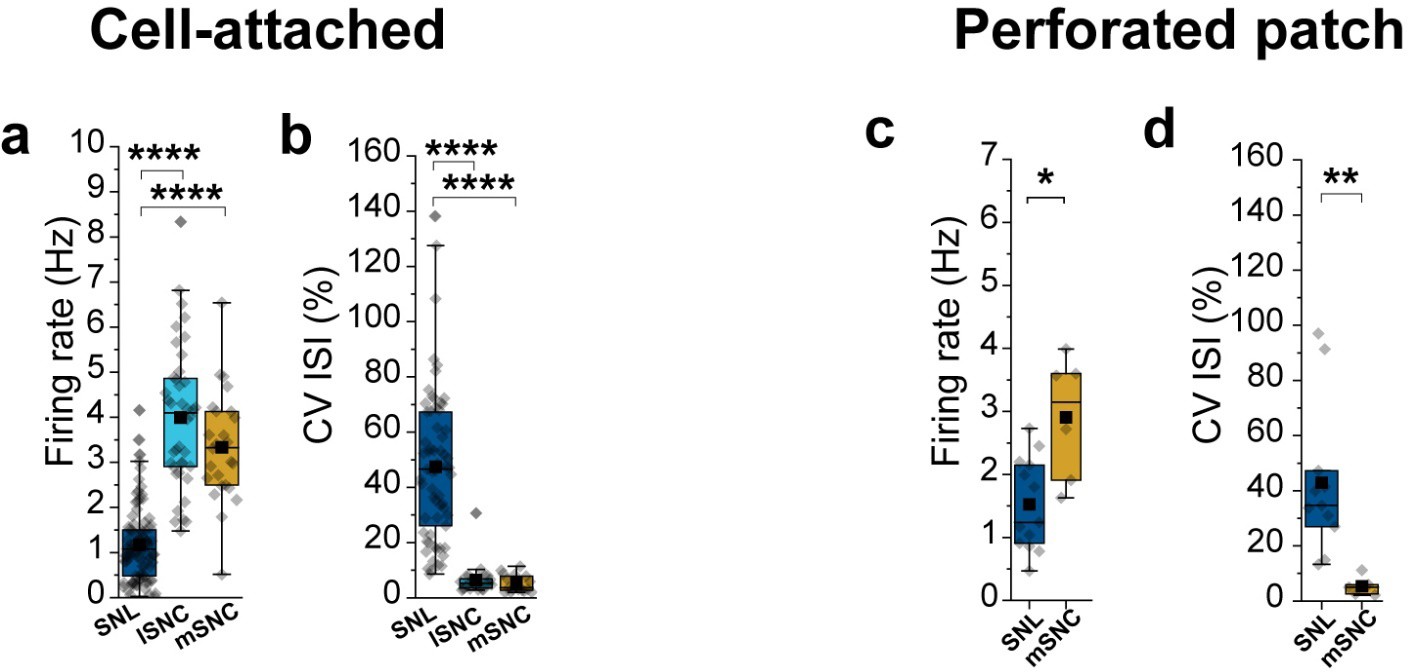


**Supplementary Figure 5 I Dopaminergic Neurons in Substantia Nigra Pars Lateralis display slow pacemaking and irregular firing rates in both cell-attached and perforated-patch recordings.** Bar plots with means showing firing rate and CV ISI of DANs obtained from electrophysiology recordings in brain slices from DAT-Cre Ai9, Calb-Cre DAT-Flp Ai65 and VGluT2-Cre DAT-Flp Ai65 mice. **a**, Cell-attached Firing rate; SNL, n = 90, 1.17 ± 0.09 Hz; lSNc, n = 40, 3.99 ± 0.25 Hz; mSNC, n = 26, 3.33 ± 0.24 Hz; SNL vs lSNC, p = 8.74 x 10^-17^, SNL vs mSNC, p = 1.53 x 10^-14^, two-sided unpaired Mann-Whitney). **b**, Cell-attached CV ISI; SNL, n = 65, 47.44 ± 3.39 %; lSNc, n = 25, 6.42 ± 1.08 %; mSNC, n = 17, 5.28 ± 0.75 %; SNL vs lSNC, p = 7.76 x 10^-19^, SNL vs mSNC, p = 2.35 x 10^-16^, two-sided unpaired Mann-Whitney). **c**, Perforated-patch Firing rate; SNL, n = 13, 1.52 ± 0.20 Hz; mSNC, n = 6, 2.90 ± 0.40 Hz; SNL vs mSNC, p = 0.017, two-sided unpaired Mann-Whitney). **d**, Perforated-patch CV ISI; SNL, n = 13, 55.18 ± 13.55 %; mSNC, n = 6, 11.44 ± 6.23 %; SNL vs mSNC, p = 0.005, two-sided unpaired Mann-Whitney). Box whiskers represent 25-75% percentiles, solid squares are mean value, horizontal box lines represent medians. ******p <* 0.05, *******p <* 0.01, ********p <* 0.001, *********p <* 0.0001.


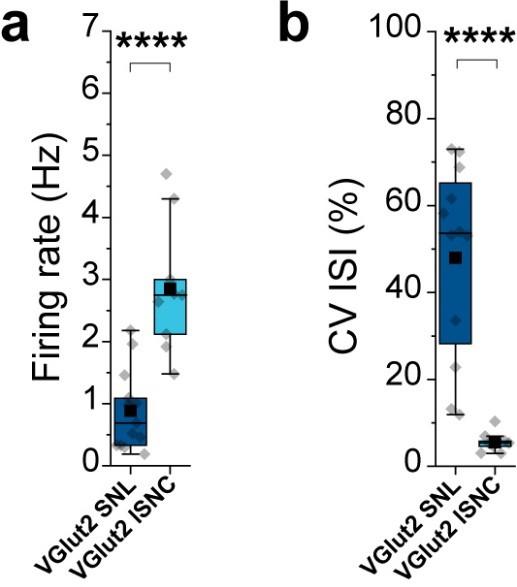


**Supplementary Figure 6 I VGluT2-positive Dopaminergic Neurons in Substantia Nigra Pars Lateralis and Substantia Nigra Pars Compacta represent two different neuronal populations.** Bar plots with means showing firing rate and CV ISI of DANs obtained from whole-cell recordings in brain slices from VGluT2-Cre DAT-Flp Ai65 mice. **a**, Cell-attached Firing rate (SNL, n = 13, 0.88 ± 0.18 Hz; lSNc, n = 9, 2.85 ± 0.35 Hz; SNL vs lSNC, p = 7.64 x 10^-5^, two-sided unpaired Mann-Whitney). **b**, Cell-attached CV ISI (SNL, n = 12, 47.96 ± 6.39%; lSNc, n = 9, 5.55 ± 0.73 %; SNL vs lSNC, p = 6.80 x 10^-6^, two-sided unpaired Mann-Whitney). Box whiskers represent 25-75% percentiles, solid squares are mean value, horizontal box lines represent medians. ******p <* 0.05, *******p <* 0.01, ********p <* 0.001, *********p <* 0.0001.


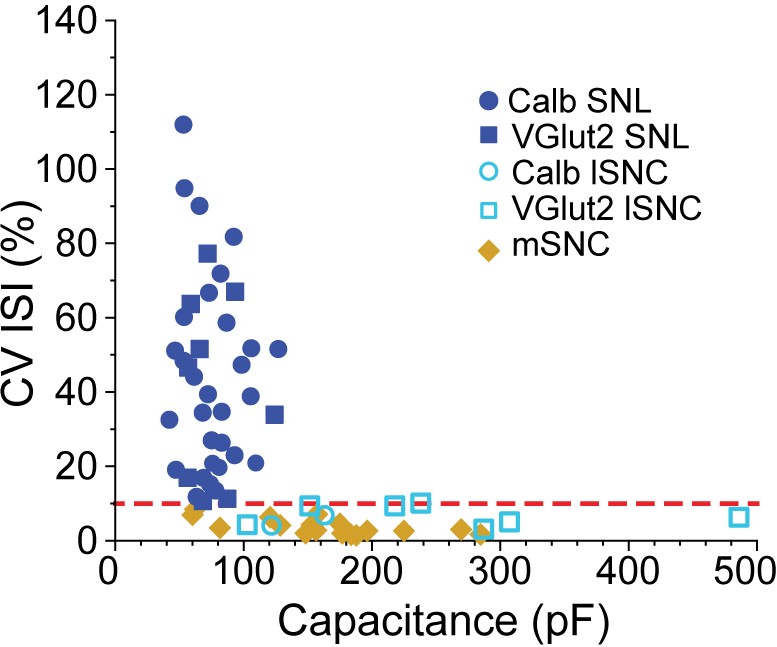


**Supplementary Figure 7 I Calb-positive and VGluT2-positive Dopaminergic Neurons in Substantia Nigra Pars Lateralis show strong correlation between firing irregularity and somatic size.** Scatter graph showing correlation between coefficient of variation of interspike interval (CV ISI) and capacitance (pF). Data obtained from whole-cell patch clamp recordings in DANs from Calb-Cre DAT-Flp Ai65, VGluT2-Cre DAT-Flp Ai65, DAT-Cre Ai9 mice and TH-GFP mice. Dotted red showing that, virtually, all SNc DANs have a CV ISI equal or lower than 10%.


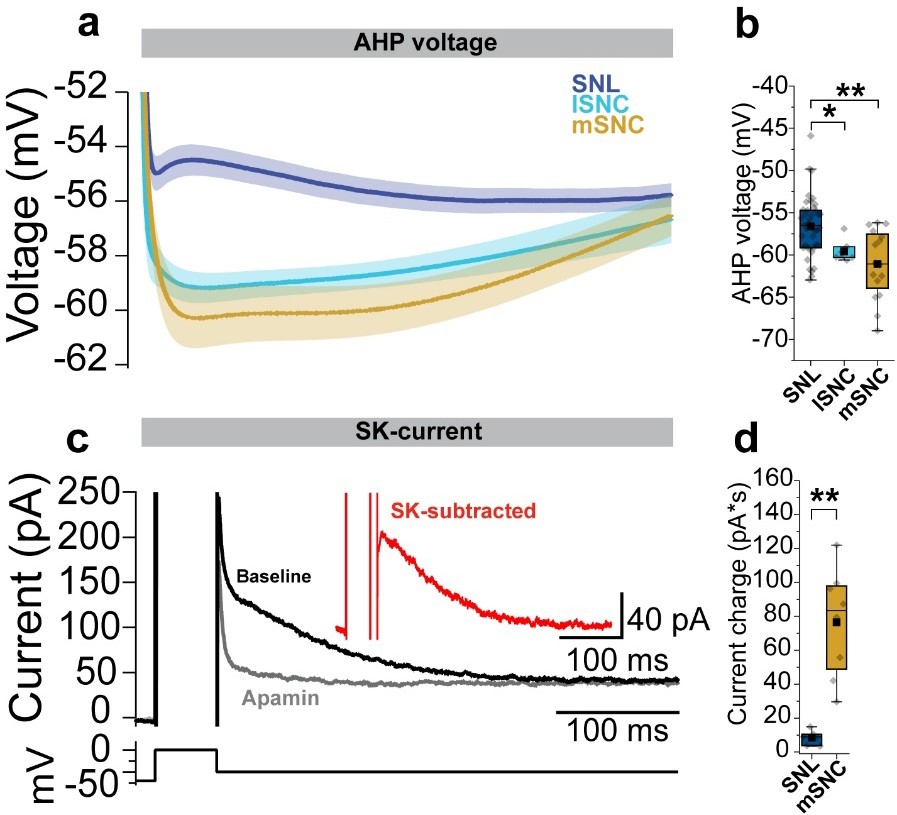


**Supplementary Figure 8 I Dopaminergic Neurons in Substantia Nigra Pars Lateralis display depolarized after-hyperpolarization voltages and smaller SK currents compared to the ones in Substantia Nigra Pars Compacta. a**, Comparison of AHP voltage during whole-cell patch-clamp recordings in SNL (blue), lSNc (cyan) and mSNc (ochre) DANs from DAT-Cre Ai9 and TH-GFP mice. **b**, Bar plots showing that average AHP voltage minima for SNL DANs are significantly smaller compared to lSNc and mSNc DANs (Average AHP minimum; SNL, n = 41, -56.61 ± 0.55 mV; lSNc, n = 7, - 59.58 ± 0.49 mV; mSNc, n = 16, 61.07 ±1.00 mV; SNL vs lSNc, p = 0.012; SNL vs mSNc, p = 4.62 x 10^-4^, two-sided unpaired Mann-Whitney). **c**, The AHP is significantly affected by small-conductance calcium-activated potassium currents (SK). A more depolarized AHP for SNL DANs suggests they have lower levels of SK channels. Thus, we performed whole-cell voltage clamp recordings to estimate SK currents in SNL and SNc DANs from DAT-Cre Ai9 and TH-GFP mice. **d**, Bar plots showing that average current charges for SNL are significantly lower compared to mSNc DANs (Current charge; SNL, n = 6, 8.42 ± 1.76 pA*sec; mSNc, n = 8, 76.5 ±11.13 pA*sec; p = 0.0022, two-sided unpaired Mann-Whitney). Box whiskers represent 25-75% percentiles, solid squares are mean value, horizontal box lines represent medians. ******p <* 0.05, *******p <* 0.01, ********p <* 0.001, *********p <* 0.0001.


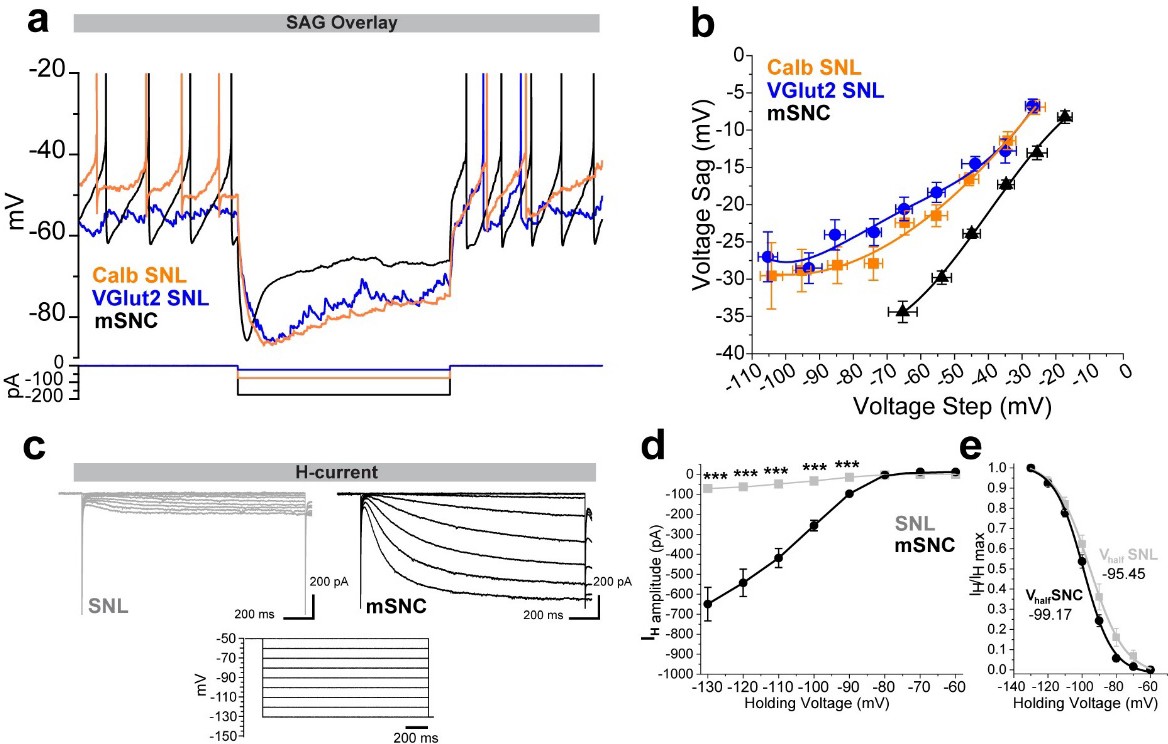


**Supplementary Figure 9 I Dopaminergic Neurons in Substantia Nigra Pars Lateralis display milder SAG voltage and HCN currents compared to the ones in Substantia Nigra Pars Compacta. a**, Whole-cell recordings showing SAG overlay for SNL (orange, blue) and mSNC (black) DANs from Calb-Cre DAT-Flp Ai65, VGluT2-Cre DAT-Flp Ai65 and DAT-Cre Ai9 mice. **b**, Voltage dependence of SAG in SNL and mSNc DANs from same mice in a. **c**, DANs SAG is mainly generated by HCN channels. Here, we isolated HCN conductances at decreasing voltages (bottom) in SNL (top left, grey) and mSNc (top right, black) DANs from VGluT2-Cre DAT-Flp Ai65 and DAT-Cre Ai9 mice. **d**, Voltage dependence of HCN current amplitude (I_H_) showing that SNL DANs display significantly lower HCN currents compared to mSNc DANs (max current amplitude; SNL, n = 8, - 70.8 ± 11.60 pA; mSNc, n = 7, - 649.07 ± 84.09; p = 3.1 x 10^-4^, two-sided unpaired Mann-Whitney). **e**, Plot showing I_H_ activation voltage showing similar profiles for SNL and mSNc DANs. ******p <* 0.05, *******p <* 0.01, ********p <* 0.001, *********p <* 0.0001.


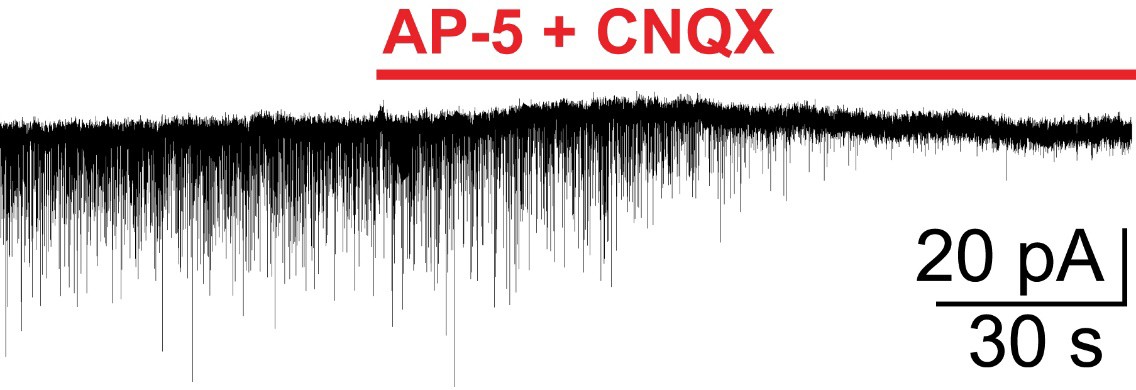


**Supplementary Figure 10 I Synaptic currents in Substantia Nigra Pars Lateralis Dopaminergic Neurons are largely excitatory.** Representative voltage clamp recording from a SNL DAN in a VGluT2-Cre mouse. Neuron was held at – 70 mV. Note the suppression of virtually all post-synaptic currents (PSCs) upon bath application of D-AP5 (50 μM) and CNQX (20 μM).

**Supplementary Figure 11 I a**, Coronal section indicating location of CTB647 injection in SNL (red). **b-d,** Coronal sections indicating retrogradely-labeled neurons that project to SNL projecting shown in different brain regions. Colored boxes (yellow/cyan) represent magnification of subsections. **b,** V1 = primary visual cortex, V2L = secondary visual cortex lateral area, TeA = temporal association cortex, SC = superior colliculus, VLPAG, = ventrolateral periaqueductal gray, PnO = pontine reticular nucleus, oral. **c**, AuD = auditory cortex, Au1 = primary auditory cortex, AuV = secondary auditory cortex ventral, EcT = ectorhinal cortex, V2M = secondary visual cortex medial area, RSD = retrosplenial dysgranular cortex, Thal = thalamus, ZI = zona incerta, CP = cerebral peduncle. **d**, AID = agranular insular cortex dorsal, AIV = agranular insular cortex ventral, DI = dysgranular insular cortex, GI = granular insular cortex, Ins = insular cortex, Pir = piriform cortex, CPu = caudate/putamen, M1 = primary motor cortex, S1 = primary somatosensory cortex, S2 = secondary somatosensory cortex. Created in BioRender. Sansalone, L. (2025) https:// BioRender.com/7e6etiy.


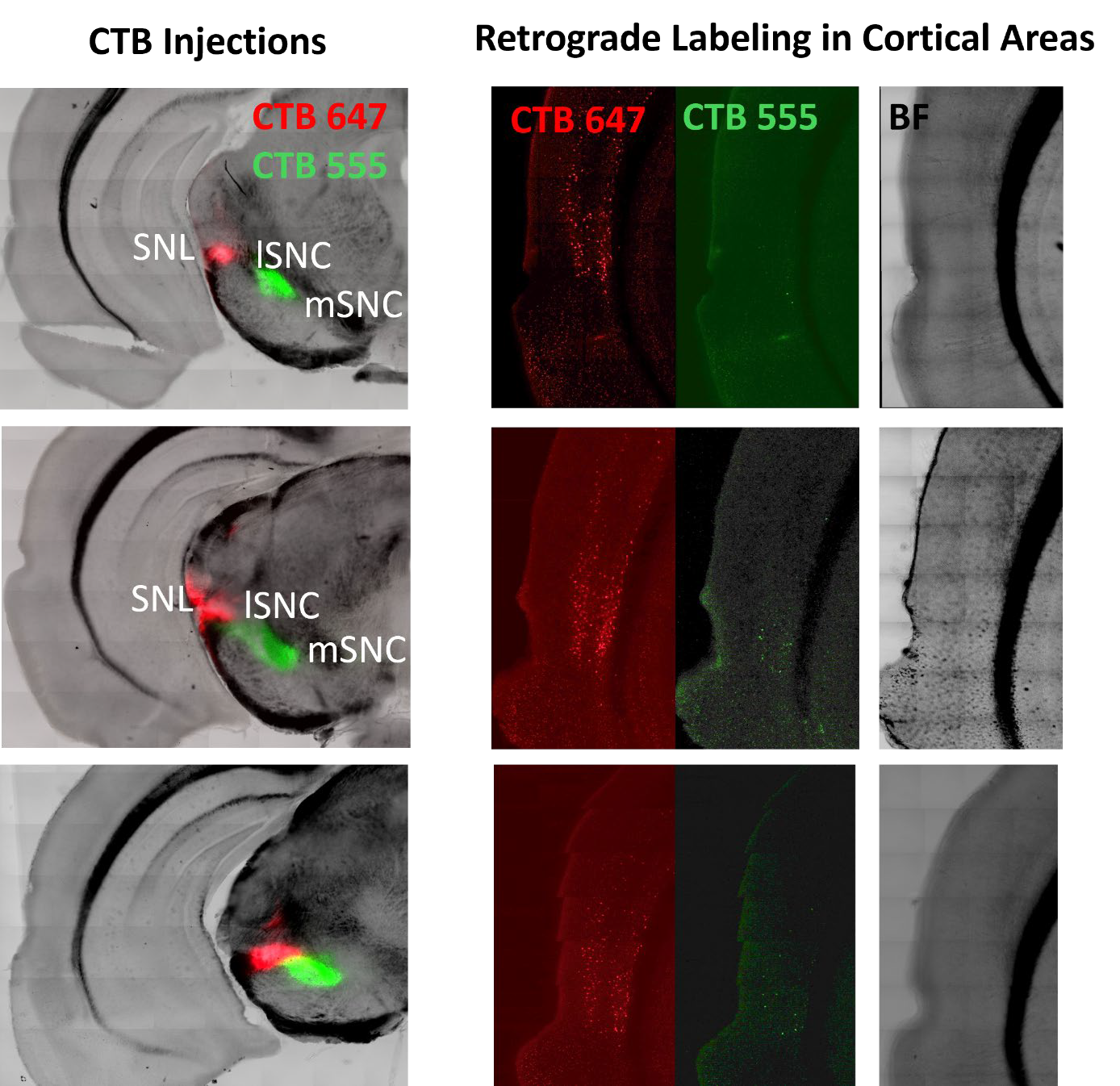


**Supplementary Figure 12 I Auditory Association Cortex innervates exclusively the Substantia Nigra Pars Lateralis but not Pars Compacta. Left**, confocal images showing progressive coronal sections from a C57BL/6J mouse that was injected in SNL and lSNc with CTB555 (green) and CTB647 (red), respectively. **Right**, confocal images showing retrogradely labeled neurons in layers 2/3 and 5 of auditory cortex (EcT/TeA/AuV) from SNL injections (red). Note that retrograde labeling from lSNc injection (green) is virtually absent, and the sparse neurons shown in green likely represent labeling through viral spread of CTB555 injection into SNL.
